# COVID-19 case management strategies: what are the options for Africa?

**DOI:** 10.1186/s40249-021-00795-7

**Published:** 2021-03-17

**Authors:** Joy Luba Lomole Waya, David Ameh, Joseph Lou K. Mogga, Joseph F. Wamala, Olushayo Oluseun Olu

**Affiliations:** World Health Organization COVID-19 Preparedness and Response Team, Juba, Republic of South Sudan

**Keywords:** COVID-19, Case management, Strategies, Africa

## Abstract

The ongoing coronavirus disease 2019 (COVID-19) pandemic has put a strain on health systems globally. Although Africa is the least affected region to date, it has the weakest health systems and an exponential rise in cases as has been observed in other regions, is bound to overwhelm its health systems. Early detection and isolation of suspected and confirmed COVID-19 cases are pivotal to the prevention and control of the pandemic. The World Health Organization (WHO) recommends that all laboratory-confirmed cases should be isolated and treated in a health care facility; however, where this is not possible due to the health system capacity, patients can be isolated in re-purposed facilities or at home. An already very apparent future challenge for Africa is facility-based isolation of COVID-19 cases, given the already limited health infrastructure and health workforce, and the risk of nosocomial transmission. Use of repurposed facilities requires additional resources, including health workers. Home isolation, on the other hand, would be a challenge given the poor housing, overcrowding, inadequate access to water and sanitation, and stigma related to infectious disease that is prevalent in many African societies. Conflict settings on the continent pose an additional challenge to the prevention and control of COVID-19 with the resultant population displacements in overcrowded camps where access to social services is limited. These unique cultural, social, economic and developmental differences on the continent, call for a tailored approach to COVID-19 case management strategies. This article proposes three broad case management strategies based on the transmission scenarios defined by WHO, and the criteria and package of care for each option, for consideration by policy makers and governments in African countries. Moving forward, African countries should generate local evidence to guide the development of realistic home-grown strategies, protocol and equipment for the management of COVID-19 cases on the continent  .

## Background: challenges of managing COVID-19 cases in Africa

The ongoing coronavirus disease 2019 (COVID-19) pandemic has severely disrupted global social, economic and cultural systems, and overwhelmed even the most resilient of health systems. Africa is currently one of the least affected regions by the pandemic with 1,716,697 cases and 37,741 deaths as of 20 December 2020 [1]. However, experiences from the other regions and modeling projections from several sources suggest the possibility of a resurgence in the number of cases if African governments do not sustain ongoing response actions [2]. Unfortunately, the continent faces unique political, socio-cultural and economic challenges which constrain its capacity to mount the required COVID-19 prevention and control interventions.

Early detection, isolation and management of confirmed COVID-19 cases are critical strategies for prevention and control of the disease. Other strategies include, among others, syndromic surveillance (to identify and test suspected cases at points of entry into countries, public places and health facilities), and prevention of virus shedding into the environment through respiratory hygiene, regular hand washing with soap and water or hand sanitizers which contain at least 60% alcohol. Social distancing is also vital to prevent contact with infected persons as well as avoidance of touching potentially contaminated surfaces, eyes, nose and mouth with contaminated hands. With the risk of transmission of the severe acute respiratory syndrome coronavirus 2 (SARS-CoV-2) by asymptomatic and pre-symptomatic infected individuals who potentially shed the virus into the environment, the importance of these preventive measures to control the transmission of COVID-19 cannot be overemphasized [3–5].

The World Health Organization (WHO) recommends that all laboratory-confirmed cases should be isolated and treated in a health care facility. However, in cases where it is not possible to isolate all cases in a health care facility, groups with the highest risk of poor outcomes should be prioritized. These include severe and critical cases, and patients with mild disease with co-morbidities or other risk factors such as advanced age. WHO made further recommendations on the criteria for selecting patients and requirements for home-based care and care in non-traditional healthcare facilities such as re-purposed hotels and stadia [6]. The United States Centers for Disease Control (CDC) has also provided similar guidance on the criteria and requirements for home care [7].

The weak health system and poor socio-economic development which is prevalent in most African countries are major obstacles for achievement of effective home and facility based-care of COVID-19 cases as recommended by WHO. Several African countries such as South Africa, Ethiopia, Nigeria and Algeria have recorded large COVID-19 outbreaks which overwhelmed their already weak health systems due to inadequate number of health care workers, lack of intensive care services, appropriate medical equipment and weak essential medicine supply chain systems. Furthermore, these large outbreaks can draw vital resources from provision of other basic healthcare services such as childhood immunization, prevention and control of prevalent diseases such as malaria, tuberculosis and HIV/AIDs, as was observed in South Africa early in the course of the outbreak [8]. This also increases the risk of infection of health care workers and contributes to the transmission of SARS-CoV-2 in health care facilities and among communities given that nosocomial transmission has played an important role in the early transmission of other emerging viral infections such as the Middle East respiratory syndrome coronavirus (MERS-CoV) and the severe acute respiratory syndrome (SARS) [9, 10].

Conversely, the high population density, poor housing, overcrowding, poor infrastructure and inadequate access to water and sanitation which are prevalent in many African communities have been major constraints for effective home-based care of COVID-19 cases. Furthermore, the unique socio-cultural context, beliefs and stigma associated with infectious disease on the continent is a challenge to home-based management of infectious diseases. This is compounded by the fact that most COVID-19 cases in the African context are likely to be managed at home, given the limited health system capacities and the likelihood of a high proportion of asymptomatic or mild cases who may not require hospitalization.

Additionally, conflict settings on the continent create complex humanitarian crises with massive population displacements into refugee or Internally Displaced Persons (IDPs) camps which are often overcrowded with inadequate access to social services thereby challenging effective COVID-19 prevention and control strategies. The foregoing presents a major dilemma for the effective management of COVID-19 cases in Africa. In view of these challenges, what then are the strategic options for effective, safe and dignified isolation and management of both symptomatic and asymptomatic COVID-19 cases on the continent? In this article, we draw experiences from the implementation of various COVID-19 case management strategies globally and available scientific evidence to propose case management options for resource-constrained African countries.

## Lessons learnt from the case management models used in Asia, Europe and America

Available scientific evidence is convergent on the importance of early detection and isolation of confirmed cases in the prevention and control of COVID-19 [11]. Detection is premised on a strong surveillance system that can identify suspected cases including asymptomatic and pre-symptomatic cases. However, there seem to be diverse views and experiences on the most effective strategy for patient isolation and management. In China, the emphasis was on health facility-based isolation of all COVID-19 cases due to challenges associated with enforcing strict isolation and provision of adequate medical care in home settings. Thus, the country invested in temporary hospitals called Fangcang shelter hospitals which were rapidly built by re-purposing public venues [12]. These served to isolate, triage, provide primary medical care, monitor and refer COVID-19 cases, measures which significantly contributed to the prevention and control of the pandemic. Fangcang hospitals were necessary as the bed capacity in existing hospitals was inadequate due to the rising number of cases. Furthermore, it emerged that patients isolated at home often did not adhere to isolation measures and posed a risk of infecting their family members [13]. They also faced the risk of delay in referral to hospital care in case their situation deteriorated because of inadequate clinical monitoring in a home setting. Other studies show that non-adherence to home isolation is caused by concern over the loss of income with compliance rates as low as 57% when there was no compensation for lost wages [14]. The perception of relative ‘well-being’ and lack of understanding of the high transmissibility of the disease in patients who are asymptomatic or have mild symptoms also contributes to poor adherence to home isolation measures.

Isolation in institutions such as the Fangcang shelters has significantly reduced home and community contact rates as compared to home isolation, delaying the epidemic peak and reducing the overall disease burden [15]. Re-purposed institutions also ease the burden on health facilities freeing beds for severe and critical cases. Although they are less resource-intensive, they still require the investment of additional resources and supplies, including health workers to care for admitted patients. Other drawbacks of health facility-based isolation and care include the high risk of infection of health workers in the absence of proper infection prevention and control measures. COVID-19 nosocomial outbreaks have been observed with environmental contamination including of air samples and ventilation systems, demonstrated in health care settings [16–18]. Implementation of stringent infection prevention and control (IPC) measures, isolation of suspected and confirmed cases in airborne infection isolation rooms, and active surveillance of SARS-COV-2 infections in health care settings, have been shown to minimize nosocomial transmissions [19, 20].

Hospitalization and mechanical ventilation of critically ill COVID-19 cases has also been associated with high mortality [21]. Investments made to expand facility care should, therefore, focus on interventions that are likely to benefit more patients. These include primary symptomatic care including provision of oxygen where needed and management of co-morbidities. These measures yield more positive outcomes as opposed to specialized or invasive interventions that benefit fewer patients whose prognosis is likely to be poor [22, 23].

The use of non-traditional facilities to isolate cases during epidemics is not a new concept. It was used during the response to the Ebola virus disease (EVD) outbreak in West Africa. Community Care Centers were established in re-purposed facilities or temporary structures to screen, triage, isolate and provide basic supportive treatment care for suspected EVD patients, thereby contributing to slowing community transmission [24–26]. Africa can learn from this experience in developing context-specific case management strategies for COVID-19. Some African countries such as Nigeria have successfully undertaken significant re-purposing of public venues for COVID-19 case management [27].

On the other hand, emphasis in Europe and America has been on the home-based isolation of mild to moderate cases of COVID-19. This approach focuses on the need to preserve available hospital beds, supplies and equipment for non-COVID-19 and critically ill COVID-19 cases and to reduce nosocomial infection of health workers. Home isolation in these settings, is also premised on the fact that most homes are more likely to meet the requirements for isolation; the higher standards of living, relatively smaller family size, available infrastructure to monitor home-isolated cases, e.g. through telemedicine, and a well-established referral system to facilitate access to further care when needed. Despite this, non-adherence to isolation measures is still a challenge in these settings.

Some countries in Africa, such as South Sudan adopted a similar approach where asymptomatic, mild and moderate cases without comorbidities or risk factors are isolated and managed at home, with symptomatic management for mild and moderate cases and close monitoring for any clinical deterioration. Mild and moderate cases with comorbidities, severe and critical cases are hospitalized to receive oxygen therapy and supportive care including treatment of co-infections as appropriate [28]. An analysis of the first 1330 confirmed COVID-19 cases within the first 60 days of the outbreak in the country, revealed that only 17% of cases (226 of 1330) were symptomatic with 95% of the symptomatic cases characterized as mild, only 11 of the 1330 cases (0.8%) were hospitalized, with 99% of cases managed at home and the overall case fatality rate was 1.1%. This case management approach served to reserve the already limited health infrastructure and health workforce for cases that needed hospitalization given that South Sudan has an inpatient bed capacity of only 6.5 per 10,000 population, no functional intensive care unit and a low core health workforce density of 7.6 per 10,000 population [29]. The country like most others on the continent, however, continues to face challenges with home-based care due to non-adherence to isolation measures and inappropriate conditions for home care due to larger family sizes and poor living conditions.

## Strategic options for effective management of COVID-19 cases in Africa

Based on the foregoing, we propose three broad case management options based on the transmission scenarios defined by WHO (Table 1) and criteria and package of care for each option (Table 2), for consideration by policy makers and governments in African countries. These options are by no means mutually exclusive, thus, depending on the context, it is essential to plan for all anticipated options given that the number of cases can increase exponentially within a short period. Decisions on adoption of these strategic options and their implementation hinge upon some fundamental principles to ensure:

1. Protection of the dignity, welfare and safety of patients and prevention of nosocomial infections of both patients and health care workers.
2. Availability of supporting services such as mental health and psychosocial care for both patients and health workers.
3. Selection and implementation of simple, realistic, cost-effective and sustainable case management strategies that build on existing systems and contribute to longer-term health system strengthening.
4. The use of selection criteria, requirements and package of services for all proposed strategies.
5. That case management options are context-specific, socially and culturally acceptable.
6. That there are realistic estimates of patient load during the various transmission scenarios and that this informs evidence-based forecasting of essential medicines, medical supplies and equipment.
7. That there is a realistic expectation of the type of clinical services available vis-à-vis the pre-outbreak status of the health system.
8. Appropriate risk communication messages, community engagement and participation are enhanced to address community fears and stigma.
9. That there is multi-sectoral engagement to leverage available resources across all sectors such as security, food, water and sanitation.

**Table 1:** Proposed COVID-19 case management strategies for African countries

COVID-19 transmission scenario	Objective	Proposed case management strategy and intervention
No COVID-19 transmission	Prepare for a potential outbreak	*Prepare for case management by:* Defining and reaching consensus on case management strategies, criteria, requirements and package of care Identify, map and re-purpose health facilities that will be used for case management Identify and map public venues and facilities that can be re-purposed for case management when health facility capacity is overwhelmed Develop clinical protocol for case management Identify, train, equip and deploy staff to designated facilities Quantify, procure and stock the required essential medicines, medical supplies and equipment Establish a COVID-19 patient referral system Establish COVID-19 triage desk in all major healthcare facilities Define other requirements such as security, food, water, shelter, etc.
Sporadic or clustered transmission of COVID-19 cases	Stop transmission and provide appropriate care	*Implement health facility-based case management strategy as part of outbreak containment measure to prevent further spread of the disease and save lives as follows:* Intensification of COVID-19 triage in all health facilities Intensified active case search, timely detection and diagnosis of cases Isolation of all suspected and confirmed cases of COVID-19 in designated health facilities (re-purposed facilities may be used to isolate suspected or asymptomatic cases) Implementation of clinical protocol; supportive care for mild cases and specialized care for moderate, severe and critical cases where feasible Ensure continuation of basic health care services for other common ailments
Widespread community transmission of COVID-19 with a huge case load	Slow transmission and provide appropriate care	*Implement a combination of both home and health facility-based case management strategies as follows:* Intensification of COVID-19 triage in all health facilities Intensified active case search, timely detection and diagnosis of cases Isolation of all suspected and confirmed, asymptomatic, mild and moderate cases without co-morbidities or high risk in home settings provided all the criteria for home-based care are met Provide health facility care for all cases with existing co-morbidity, severe and critical cases Consider re-purposed facilities and community care centers for isolation of suspected, asymptomatic, mild and moderate cases who cannot be isolated at home for various reasons Implementation of clinical protocol; supportive care for uncomplicated mild and moderate cases and specialized care for complicated moderate, severe and critical cases where feasible Ensure continuation of basic health care services for other common ailments

**Table 2:** Criteria and package of care for management of COVID-19 cases in Africa

Strategy	Criteria	Package	Requirements
Health facility	Severe and critical cases Mild and moderate cases with risk factors for poor outcomes (co-morbidities, age > 60 years) All confirmed cases if resources allow All suspected cases if resources allow	Medical care as appropriate Supportive treatment Treat co-infections and co-morbidities Oxygen therapy Mechanical ventilation for critical cases Psychosocial support for patients and health workers Food, water and other necessities	Dedicated trained COVID-19 health workers/teams Adequate Personal Protective Equipment for health workers Oxygen generation plant/cylinders Ventilators and other intensive care supplies/consumables Critical care doctors and nurses Essential medicines
Re-purposed facility	Mild and moderate cases with no risk factors Asymptomatic cases (if resources allow) All suspected cases (if resources allow)	Basic medical care as appropriate Supportive treatment Treat co-infections Monitor for clinical deterioration to institute referral Psychosocial support for patients and health workers Food, water and other necessities Social engagement activities	Dedicated trained COVID-19 health workers/teams (community health workers can be trained) Basic essential medicines that can be administered by community health workers Adequate Personal Protective Equipment for health workers Access to ambulance services for referral to a health facility in case of clinical deterioration Recreational facilities
Home-based care	If there is no capacity for isolation in designated facilities, home isolate: Suspected cases Asymptomatic cases Mild and moderate cases with no risk factors	Home based care kit with essential IPC supplies (e.g. soap, disinfectant, medical masks) and medications for supportive treatment such as antipyretics, as adapted for the country context Regular communication with community health workers to monitor for any clinical deterioration (this can be done by phone or physical visits at scheduled intervals) Scheduled visits by community health workers to monitor adherence to IPC measures Psychosocial support for patients and caregivers	Assessment of the home by a health care worker to assess its suitability for home-based care Training of caregivers on infection prevention and control measures Community engagement to address stigma Legal enforcement to ensure adherence to isolation measures Compensation for loss of income e.g. by provision of food or non-food items or cash Access to ambulance services for referral to a health facility in case of clinical deterioration

## Conclusions

Given its unique sociocultural and socioeconomic context and fragile health system, there are no simple options neither does one size-fit-all in the management strategies of COVID-19 cases in Africa. African countries will therefore, have to define home-grown strategies which are socially and culturally acceptable and realistic given its level of socioeconomic development and health system maturity. In doing so, the health policymakers in African countries should review and adopt relevant scientific evidence, lessons, practical experiences and guidelines from other regions to define its criteria and decision-making tool for selecting the appropriate COVID-19 case management strategies, ensuring a balance of science with sociocultural and socioeconomic realities. This article provides broad guidance in this regard.

The lessons learnt and capacities built from managing other infectious diseases outbreaks such as EVD should serve as guidance for COVID-19 case management. Furthermore, it is necessary to apply phased and step-wise approaches to scale up COVID-19 case management in a realistic manner; in other words, it is critical to focus on what is possible and plausible given the context. Furthermore, African countries should use the opportunity of the ongoing COVID-19 pandemic response to strengthen their health systems in general and to improve their capacity for the management of patients that require intensive care in particular. Specifically, appraisal of the competency of health care workers, capacity building on COVID-19 clinical management and availability of appropriate equipment for management of cases and protection of healthcare workers is critical. Finally, we recommend that African governments and scientists should strengthen national capacities for the generation of local evidence which could guide the development of home-grown case management strategies, protocols and equipment for the management of COVID-19 cases on the continent.

## Data Availability

Not applicable.

## Abbreviations

CDC: The United States Center for Disease Control and Prevention
COVID-19: Coronavirus disease 2019
EVD: Ebola virus disease
HIV/AIDS: Human immunodeficiency virus/acquired immunodeficiency syndrome
IDPs: Internally displaced persons
IPC: Infection prevention and control
MERS-CoV: Middle East respiratory syndrome coronavirus
SARS: Severe acute respiratory syndrome
SARS-CoV-2: Severe acute respiratory syndrome coronavirus 2
WHO: World Health Organization
